# Total average diastolic longitudinal displacement by colour tissue doppler imaging as an assessment of diastolic function

**DOI:** 10.1186/s12947-016-0083-2

**Published:** 2016-09-17

**Authors:** Martina Chantal de Knegt, Tor Biering-Sørensen, Peter Søgaard, Jacob Sivertsen, Jan Skov Jensen, Rasmus Møgelvang

**Affiliations:** 1 Herlev and Gentofte Hospital, Department of Cardiology, Faculty of Health Sciences, University of Copenhagen, Copenhagen, Denmark; 2 Department of Cardiology, Centre for Cardiovascular Research, Aalborg University Hospital, Aalborg, Denmark; 3 Rigshospitalet, Department of Cardiology, Faculty of Health Sciences, University of Copenhagen, Copenhagen, Denmark

**Keywords:** Echocardiography, Colour tissue Doppler imaging, Diastolic function, Diastolic longitudinal displacement

## Abstract

**Background:**

The current method for a non-invasive assessment of diastolic dysfunction is complex with the use of algorithms of many different echocardiographic parameters. Total average diastolic longitudinal displacement (LD), determined by colour tissue Doppler imaging (TDI) via the measurement of LD during early diastole and atrial contraction, can potentially be used as a simple and reliable alternative.

**Methods:**

In 206 patients, using GE Healthcare Vivid E7 and 9 and Echopac BT11 software, we determined both diastolic LD, measured in the septal and lateral walls in the apical 4-chamber view by TDI, and the degree of diastolic dysfunction, based on current guidelines. Of these 206 patients, 157 had cardiac anomalies that could potentially affect diastolic LD such as severe systolic heart failure (*n* = 45), LV hypertrophy (*n* = 49), left ventricular (LV) dilation (*n* = 30), and mitral regurgitation (*n* = 33). Intra and interobserver variability of diastolic LD measures was tested in 125 patients.

**Results:**

A linear relationship between total average diastolic LD and the degree of diastolic dysfunction was found. A total average diastolic LD of 10 mm was found to be a consistent threshold for the general discrimination of patients with or without diastolic dysfunction. Using linear regression, total average diastolic LD was estimated to fall by 2.4 mm for every increase in graded severity of diastolic dysfunction (β = −0.61, *p-value* <0.001). Patients with LV hypertrophy had preserved total average diastolic LD despite being classified as having diastolic dysfunction. Reproducibility of LD measures was acceptable.

**Conclusions:**

There is strong evidence suggesting that patients with a total average diastolic LD under 10 mm have diastolic dysfunction.

**Electronic supplementary material:**

The online version of this article (doi:10.1186/s12947-016-0083-2) contains supplementary material, which is available to authorized users.

## Background

There is no simple means of reliably diagnosing left ventricular (LV) diastolic dysfunction: The current method for a non-invasive assessment of diastolic function requires the use of algorithms primarily based on a pulsed Doppler measurement of early mitral inflow velocity (E) and a colour tissue Doppler imaging (TDI) assessment of early mitral annular velocity (e’), thereby giving an indirect assessment of LV filling pressures (E/e’), as determined by the Bernouili principle and Laplace law [1]. Other parameters include an assessment of early and atrial mitral inflow patterns (E/A), deceleration time (DT), and left atrial (LA) volume [2]. Using these parameters, LV diastolic dysfunction is traditionally classified as grade 1: abnormal relaxation; grade 2: pseudonormal relaxation, or grade 3: restrictive filling pattern.

Even though it is agreed that no single current echocardiographic measure is sufficient for a diagnosis of LV diastolic dysfunction, the use of algorithms of many different echocardiographic parameters is also problematic as a situation of “one size fits all” arises. Additionally, the fundamental use of velocity based parameters in these algorithms is deficient as the use of velocities as a determinant of diastolic function only takes one snapshot of imaging into consideration, thereby making an accurate depiction of severity difficult to obtain, especially in patients with atrial fibrillation. Lastly, the echocardiographic parameters used in these algorithms are only estimates of LV filling pressures and are subject to limitations of the imaging technique, such as angle dependency, sample volume, and tethering artifacts as well as to shortcomings inherent to derivation of pressures from inflow or re-extension signals [1].

Furthermore, the reliability, feasibility, and practical utility of the individual markers of diastolic dysfunction have been questioned and concordance has been shown to be poor [3, 4]. With regards to e’, septal and lateral measurements differ and it has, therefore, been recommended that averages be used [5]. e’ can also be reduced inaccurately by mitral annular calcification, surgical rings, or prosthetic valves [1]. With regards to E/e’, pulsed Doppler velocities have been shown to overestimate colour TDI velocities, thereby, potentially leading to error in the assessment of LV diastolic function [6]. The use of E/e’ has also been questioned in patients with hypertrophic and dilated cardiomyopathy and in decompensated patients with resynchronization therapy [7, 8]. Moreover, E/e’ >15 is associated with an elevated LV filling pressure and E/e’ <8 is evidence of normal filling pressure - there is, therefore, a big gap (between E/e' 8-15) for which additional investigations are required to obtain a LV filling pressure estimate [1]. The use of E/A and DT is also problematic as both measures are bimodal and a situation with pseudonormalisation occurs, making it difficult to discern between a patient with normal diastolic function and a patient with a relatively severe grade of diastolic dysfunction.

Our group has previously shown that a combined assessment of both early and late diastolic velocities by colour TDI is a strong prognostic marker of acute myocardial infarction, heart failure, and cardiovascular death in the general population [9]. This indicates that an assessment of both early and late diastole is important for a meaningful assessment of true LV diastolic function. Total diastolic longitudinal displacement (LD) calculated via the integration of the early (E) and atrial (A) velocity waves, obtained by colour TDI, is a potentially new measurement of diastolic function that avoids the major drawbacks associated with the current velocity based assessment, namely an independent measurement that has a linear relationship with the degree of diastolic dysfunction, i.e. non-biphasic, and a measurement that takes the whole diastolic process into consideration, i.e. not just a snapshot of imaging.

The aims of this study are to investigate: 1) The potential of colour TDI determined total diastolic LD in the determination of diastolic function in the healthy and dysfunctional heart, 2) The ability of total diastolic LD to predict adverse outcomes, 3) To assess reproducibility of total diastolic LD.

## Methods

### Data source

The Department of Cardiology, Herlev and Gentofte Hospital, University of Copenhagen performs routine echocardiograms according to a standardised protocol and these echocardiograms are stored on a local server.

### Study population

A total of 486 consecutive patients were retrieved from the echocardiogram database. To obtain a representation of structural heart disease (no cardiac abnormalities; severe systolic heart failure; LV hypertrophy; LV dilation; and mitral regurgitation), the echocardiograms were analysed in accordance with the following criteria:

Inclusion criteria:

- No cardiac abnormalities: Eyeball LVEF >50 % and normal cardiac dimensions.
- Severe systolic heart failure: Eyeball LVEF <30 %.
- LV hypertrophy: LV mass/body surface area ≥113 g/m^2^ for women and ≥131 g/m^2^ for men.
- LV dilation: LV internal dimension at end-diastole/body surface area ≥3.8 cm/m^2^ for women and ≥3.7 cm/m^2^ for men.
- Mitral regurgitation: ≥grade 2.

Exclusion criteria:

- Overlapping diseases of the above mentioned, for example, mitral regurgitation in a patient with LV hypertrophy. Exceptions: patients with a dilated LV, hypertrophic LV, or mitral regurgitation may have had a LVEF under 30 %.
- Incomplete examinations.
- Bundle branch block.
- Atrial fibrillation.
- Unsuitability for colour TDI, i.e. indistinguishable E- and A-wave.

A total of 89 patients fulfilled inclusion criteria for “no cardiac abnormalities”; 138 patients fulfilled inclusion criteria for “severe systolic heart failure”; 117 patients fulfilled criteria for “LV hypertrophy”; 75 patients fulfilled inclusion criteria for “LV dilation”; and 67 patients fulfilled criteria for “mitral regurgitation”. After application of exclusion criteria, the final study population consisted of 206 patients, 49 of whom were classified as having normal cardiac dimensions and function. The 157 remaining patients all had heart anomalies that could potentially affect diastolic LD such as severe systolic heart failure (*n* = 45), LV hypertrophy (*n* = 49), LV dilation (*n* = 30), and mitral regurgitation (*n* = 33). Diastolic function was assessed and participants were graded as having a normal relaxation (*n* = 76), abnormal relaxation (*n* = 75), pseudonormal relaxation (*n* = 44), or restrictive filling pattern (*n* = 11).

### Echocardiographic analyses

All echocardiograms were obtained using Vivid E7 and Vivid 9 ultrasound systems (GE Healthcare, Horten Norway) and all images were stored digitally on a central server. All participants were examined with conventional two-dimensional echocardiography, m-mode, pulsed-wave TDI, colour TDI and two-dimensional strain imaging. All the echocardiographic analyses in the present study were performed de novo and offline (blinded to other clinical data) using Echopac BT11 software (GE Healthcare, Horten Norway).

#### Conventional echocardiography

LV end-diastolic dimensions (interventricular septum wall thickness (IVS), LV internal dimension (LVID), and LV posterior wall thickness (PWT)) and LA diameter were obtained from the parasternal long-axis view at the mitral valve leaflet tips. Pulsed wave Doppler was used to record mitral inflow between the tips of the mitral leaflets. LVEF was obtained using modified Simpson’s biplane method in the 4-chamber and 2-chamber view [10]. LA volume was estimated by the area-length method [10]. LV mass index was calculated as the anatomic mass (LV mass = 0.8x(1.04[(IVSd + LVIDd + PWTd)3 − LVIDd3]) + 0.6 g) [11], divided with body surface area. All measurements were acquired from the parasternal approach and measured at the level of the mitral valve at end-diastole. Relative wall thickness was calculated as (2 × LV PWT)/(LVID) and the subgroup of patients with LV hypertrophy was sub-categorized with regards to concentric or eccentric hypertrophy in accordance with current American Society of Echocardiography Committee recommendations [10]. Severity of mitral regurgitation was determined according to current guidelines [12]. All chamber quantifications are in accordance with the American Society of Echocardiography Committee recommendations [10].

#### Tissue Doppler Imaging (TDI)

**Fig. 1:**
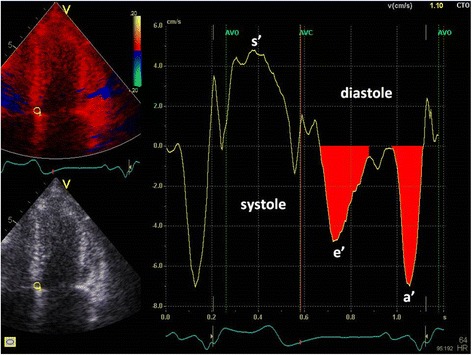
Image of colour tissue Doppler imaging with septal longitudinal displacement measurements depicted as area under the curve in early and late diastole. *s’, peak mitral annular systolic velocity; e’, peak early mitral annular diastolic velocity; a’, peak atrial mitral annular diastolic velocity; AVO, aortic valve opening; AVC, aortic valve closing*

Colour TDI loops were obtained in the apical 4-chamber view. Peak longitudinal systolic velocities (s’) as well as early diastolic (e’) and late diastolic (a’) velocities were measured in the septal and lateral wall using a 5 mm circular sample volume placed in the left ventricular myocardial just proximal to the mitral annular level. Total average systolic and diastolic LD was determined by calculating the area under the curve, using tissue tracking software, of the systolic, early and late diastolic waves, respectively, and averaging septal and lateral findings (Fig. 1). Potential L-waves between the early and late diastolic waves were not included in the assessment of total average diastolic displacement. Systolic displacement was measured as the maximal displacement between aortic valve opening and aortic valve closure and post-systolic contraction was not included in systolic displacement measurements.

Pulsed wave TDI tracings, used to measure peak myocardial velocities, were obtained with the range gate placed at the septal and lateral mitral annular segments in the 4-chamber view. The average of septal and lateral e’ by pulsed wave TDI was used to calculate E/e’.

#### M-mode

Mitral annular plane systolic excursion (MAPSE) was determined in the septal and lateral walls in the apical four-chamber view in all participants. This was done by placing the M-mode cursor in the septal and lateral borders in the mitral annular plane. In doing so, an image of either septal or lateral mitral annular displacement was obtained. MAPSE was measured from the lowest to the highest point of contraction, excluding post-systolic contractions, i.e. measurements were obtained between aortic valve opening and aortic valve closure.

#### Two-dimensional Strain Imaging (2DSI)

Using 2-dimensional speckle tracking derived strain imaging, the left ventricular myocardial wall was traced in the 4-chamber view with the basal septal and lateral segments starting at the mitral annular level. By the tracking of speckles from frame-to-frame, myocardial deformation was assessed. The average of regional measurements in a 6-segment model from the apical 4-chamber view was used as an estimate of longitudinal displacement (LD), longitudinal strain (LS) and longitudinal strain rate (LSR). Regional basal longitudinal displacement, strain, and strain rate was calculated as the average of the basal septal and lateral segments in the apical 4-chamber view.

TDI and strain measurements are reported as absolute values.

### Determination of degree of diastolic dysfunction

Patients were graded as having either normal diastolic function or abnormal diastolic function grade 1–3 according to current EAE/ASE recommendations [2].

### Prognostics study

Data on mortality (primary endpoint) was obtained using the unique personal identification number in the Central Office of Civil Registration and the Danish National Board of Health’s National Patient Registry.

### Statistical analysis

All statistical analyses were done using SPSS version 20 (SPSS, Inc, Chicago, IL). In Table 1, continuous Gaussian distributed variables and proportions were compared using Student’s *t*-test and *χ*2-test, respectively. Pearsons correlation was used to determine any associations between the degree of diastolic dysfunction and total average diastolic LD as well as between total average diastolic LD and individual parameters used in the assessment of diastolic and systolic function. Using logistic regressions, odds ratios for unadjusted and adjusted models were calculated to establish the association between peak velocities and diastolic LD and the primary endpoint (death). Interactions with the subgroup type were tested for. Bland-Altman analysis was used to evaluate intraobserver and interobserver variability and was expressed as mean difference ± 1.96 standard deviation (SD) and as coefficient of variation (CV) [13]. *P*-values <0.05 in two-sided tests were considered statistically significant.

**Table 1:** Characteristics of the patients studied

	Controls (*n* = 49)	Severe Systolic HF (*n* = 45)	LV Hypertrophy (*n* = 49)	LV Dilation (*n* = 30)	Mitral Regurgitation (*n* = 33)
Age, y	58 ± 15	65 ± 15*	69 ± 15***	76 ± 9***	71 ± 11***
Male sex, % (n)	43 (21)	93 (42)	47 (23)	57 (17)	55 (18)
Height, cm	173 ± 9	177 ± 9*	171 ± 10	167 ± 7**	171 ± 9
Weight, kg	76 ± 16	82 ± 15	73 ± 14	65 ± 14**	72 ± 15
LVIDd, cm	4.7 ± 0.6	5.6 ± 0.7***	5.2 ± 0.7**	6.8 ± 0.8***	5.0 ± 0.9
BSA, m^2^	1.9 ± 0.2	2.0 ± 0.2*	1.8 ± 0.2	1.7 ± 0.2***	1.8 ± 0.2
LVIDd/BSA, cm/m^2^	2.5 ± 0.3	2.9 ± 0.4***	2.8 ± 0.4***	4.0 ± 0.2***	2.7 ± 0.5*
LVEF, %	64 ± 7	30 ± 9***	60 ± 7*	25 ± 12***	53 ± 16***
IVS, cm	0.9 ± 0.2	0.9 ± 0.2	1.2 ± 0.2***	1.0 ± 0.2	1.0 ± 0.3
LVPWd, cm	0.9 ± 0.2	0.9 ± 0.2	1.2 ± 0.2***	1.0 ± 0.1	1.0 ± 0.3
LVM/BSA, g/m^2^	70 ± 16	102 ± 19***	130 ± 23***	171 ± 42***	103 ± 37**
DDF 0, % (n)	74 (36)	24 (11)	35 (17)	0 (0)	36 (12)
DDF 1, % (n)	22 (11)	51 (23)	39 (19)	47 (14)	24 (8)
DDF 2, % (n)	4 (2)	11 (5)	27 (13)	47 (14)	30 (10)
DDF 3, % (n)	0 (0)	13 (6)	0 (0)	7 (2)	9 (3)
E/A	1.2 ± 0.5	1.2 ± 0.8	1.1 ± 0.5	1.4 ± 1.0	1.5 ± 0.6**
DT, ms	221 ± 57	191 ± 98	236 ± 59	218 ± 66	223 ± 93
LAV, ml/m^2^	24.5 ± 9.0	33.8 ± 10.5***	39.6 ± 13.7***	45.5 ± 18.2***	46.5 ± 16.5***
E/e’	8.6 ± 3.6	12.3 ± 6.4**	12.4 ± 5.4***	17.0 ± 7.9***	19.3 ± 14.9***
Known IHD, % (n)	18 (9)	69 (31)	4 (2)	17 (5)	21 (7)

*HF* heart failure, *LV* Left ventricular, *LVIDd* LV internal dimension in diastole, *BSA* body surface area, *LVEF* LV ejection fraction, *IVS* interventricular septum, *LVPWd* LV posterior wall in diastole, *LVM* LV mass, *DDF* degree of diastolic dysfunction, *E/A* early inflow velocity/atrial inflow velocity, *DT* deceleration time, *LAV* left atrial volume, *E/e’* early inflow velocity/early diastolic tissue velocity, *IHD* ischemic heart disease

*P <0.05 compared with the control group

**P <0.01 compared with the control group

***P <0.001 compared with the control group

## Results

### Patient characteristics

Main characteristics of the study population are presented in Table 1. Parameters of advanced echocardiography are presented in Table 2. Compared to controls, patients with structural heart disease had both reduced early and active diastolic function as assessed by various echocardiographic techniques such as TDI and speckle tracking. Of the 49 patients with LV hypertrophy, 32 (65 %) had concentric hypertrophy; 17 (35 %) had eccentric hypertrophy.

**Table 2:** Advanced echocardiographic parameters within subgroups

	Controls (*n* = 49)	Severe Systolic HF (*n* = 45)	LV Hypertrophy (*n* = 49)	LV Dilation (*n* = 30)	Mitral Regurgitation (*n* = 33)
TDI					
s’, cm/s	6.2 ± 1.4	3.8 ± 1.2***	5.4 ± 1.1**	2.9 ± 1.1***	5.3 ± 2.3
e’, cm/s	−7.6 ± 2.3	−4.4 ± 1.6***	−5.9 ± 2.2***	−3.6 ± 1.7***	−5.3 ± 2.5***
a’, cm/s	−7.6 ± 1.8	−5.7 ± 2.3***	−6.8 ± 1.7*	−4.3 ± 2.0***	−5.6 ± 2.6***
e’ + a’, cm/s	−15.2 ± 2.7	−10.1 ± 2.5***	−12.7 ± 2.2***	−7.9 ± 2.7***	−11.0 ± 4.6***
TT-s’, mm	11.4 ± 2.0	6.5 ± 2.3***	10.8 ± 2.0	4.7 ± 2.5***	9.3 ± 4.3*
TT-e’, mm	7.3 ± 2.2	3.6 ± 1.8***	5.6 ± 2.1***	2.6 ± 1.2***	4.8 ± 2.6***
TT-a’, mm	5.2 ± 1.5	4.0 ± 1.7***	5.2 ± 1.5	3.1 ± 1.6***	4.2 ± 1.9**
TT-e’ + TT-a’, mm	12.6 ± 2.3	7.5 ± 2.2***	10.8 ± 2.2***	5.7 ± 1.9***	9.0 ± 4.0***
M-mode					
MAPSE, mm	12.3 ± 2.4	7.4 ± 2.1***	11.3 ± 2.0*	5.4 ± 2.2***	10.4 ± 4.1*
2D strain					
LS, %	−14.8 ± 3.8	−9.1 ± 4.5***	−12.0 ± 3.8**	−6.6 ± 4.3***	−12.1 ± 4.8**
LD, mm	15.4 ± 2.6	7.7 ± 3.0***	15.8 ± 3.0	6.1 ± 3.8***	12.7 ± 5.7*
s, cm/s	6.5 ± 1.5	4.0 ± 1.0***	5.7 ± 1.0**	2.5 ± 2.0***	5.0 ± 1.9***
e, cm/s	−6.5 ± 2.8	−3.8 ± 1.6***	−5.4 ± 2.1*	−2.3 ± 1.9***	−5.3 ± 2.7
a, cm/s	−7.4 ± 1.7	−5.3 ± 1.9***	−6.5 ± 2.0*	−4.1 ± 1.8***	−5.4 ± 2.2***
e + a, cm/s	−13.9 ± 3.2	−9.1 ± 2.3***	−11.9 ± 2.6**	−6.5 ± 3.0***	−10.7 ± 4.3**
s, s^−1^	−1.0 ± 0.3	−0.8 ± 0.2***	−0.9 ± 0.4	−0.8 ± 0.3**	−1.0 ± 0.4
e, s^−1^	1.3 ± 0.4	0.9 ± 0.5***	1.0 ± 0.3**	0.7 ± 0.4***	1.1 ± 0.4*
a, s^−1^	1.2 ± 0.4	0.9 ± 0.4***	1.1 ± 0.5	0.9 ± 0.5**	1.1 ± 0.4
e + a, s^−1^	2.5 ± 0.5	1.8 ± 0.6***	2.2 ± 0.7*	1.6 ± 0.8***	2.1 ± 0.7**

*TDI* tissue Doppler Imaging, *s’* systolic tissue velocity by TDI measured at the mitral annulus, *e’* early diastolic tissue velocity by TDI measured at the mitral annulus, *a’* late diastolic tissue velocity by TDI measured at the mitral annulus, *TT-s’* systolic displacement measured at the mitral annulus, *TT-e’* early diastolic displacement measured at the mitral annulus, *TT-a’* late diastolic displacement measured at the mitral annulus, *MAPSE* M-mode derived mitral annular plane systolic excursion, *LS* longitudinal strain by 2D strain imaging, *LD* longitudinal displacement by 2D strain imaging, *s* systolic tissue velocity/strain rate by 2D strain imaging measured at the basal septal and lateral segments, *e* early diastolic tissue velocity/strain rate by 2D strain imaging measured at the basal septal and lateral segments, *a* late diastolic tissue velocity/strain rate by 2D strain imaging measured at the basal septal and lateral segments

*P <0.05 compared with the control group

**P <0.01 compared with the control group

***P <0.001 compared with the control group

### Total average diastolic longitudinal displacement and degree of diastolic dysfunction

**Fig. 2:**
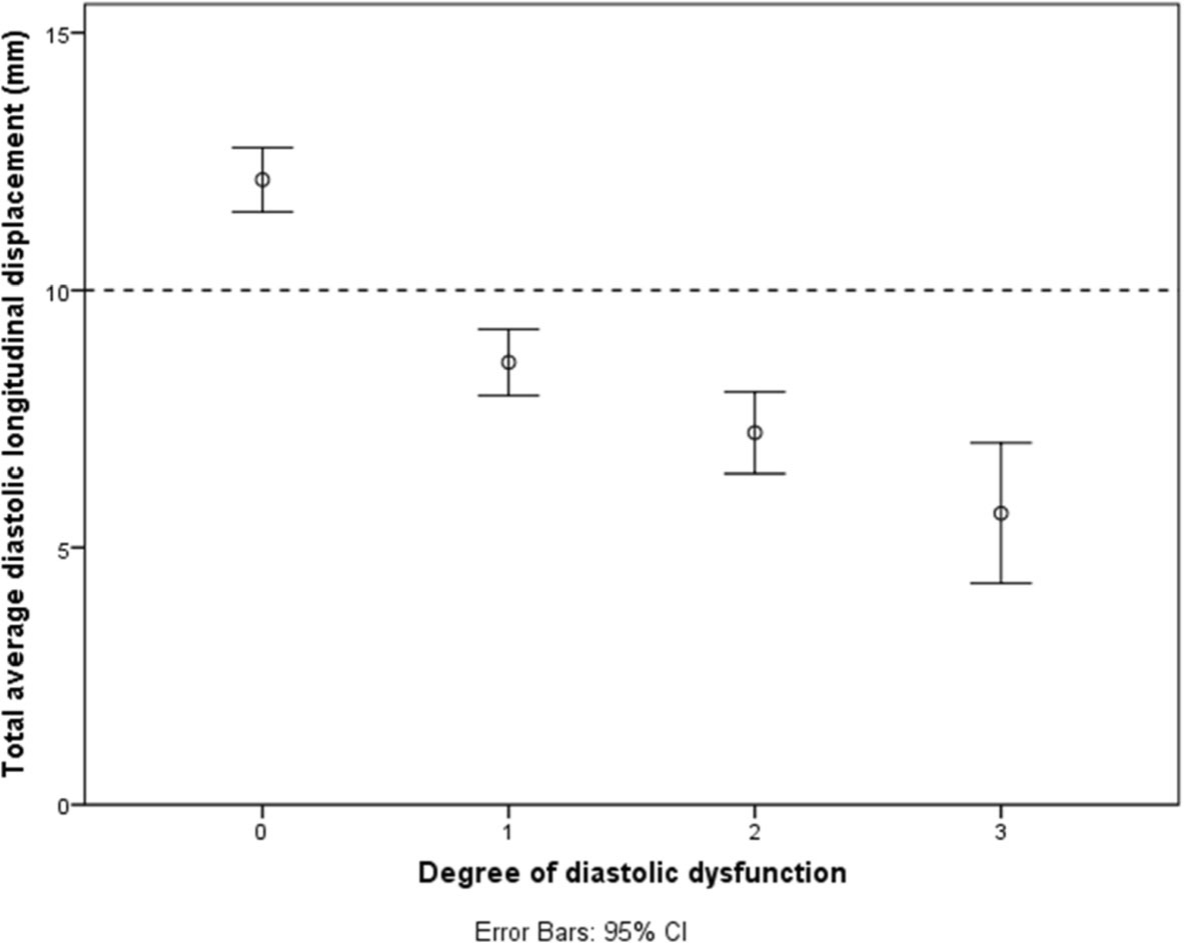
Total average diastolic longitudinal displacement and the degree of diastolic dysfunction. *HF, heart failure*

**Fig. 3:**
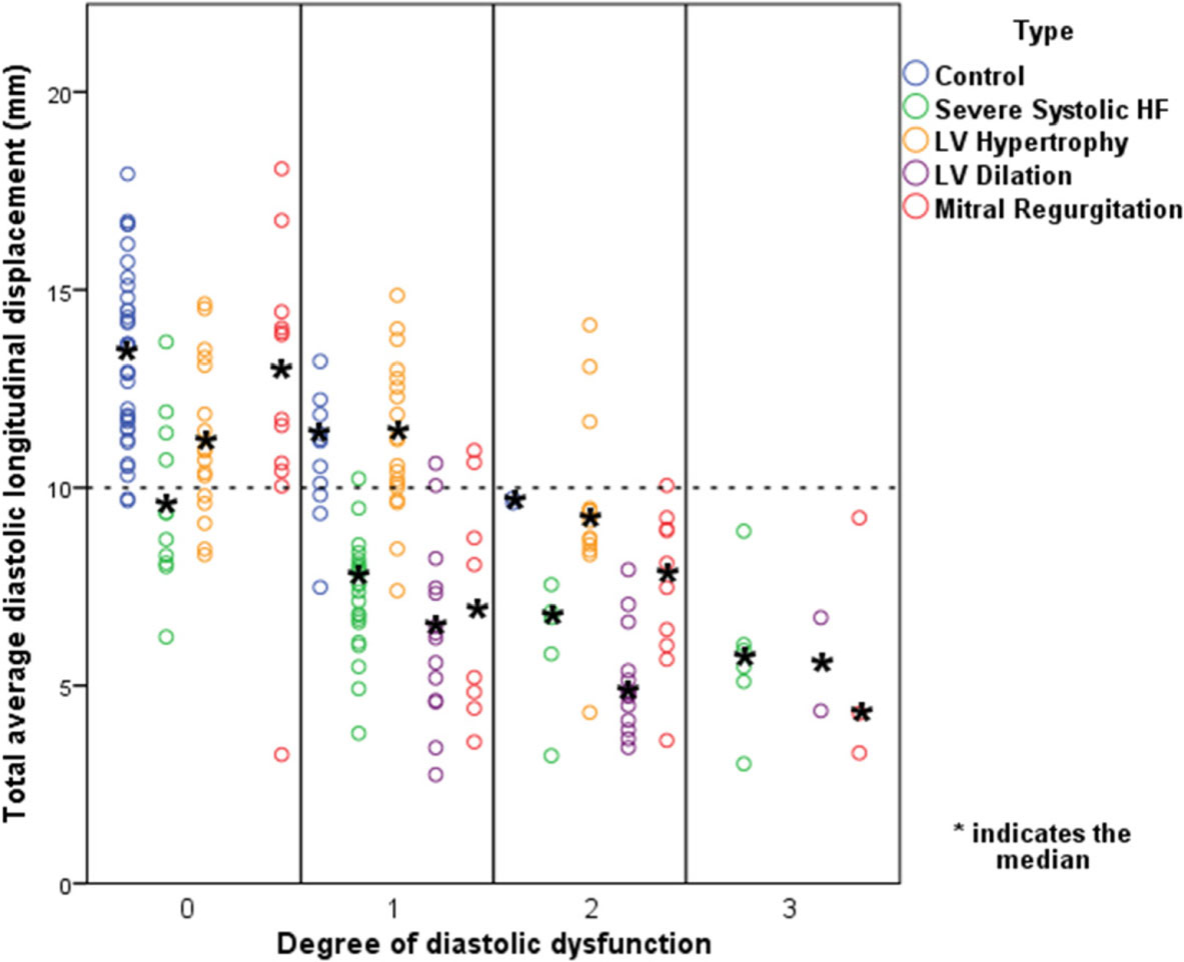
Total average diastolic longitudinal displacement in various heart conditions and the degree of diastolic dysfunction *HF, heart failure*

A linear relationship, also after adjustment for age and gender, between total average diastolic LD and the degree of diastolic dysfunction was found (Fig. 2). This linear relationship is due to a fall in TT-e’ in the mild stages of diastolic dysfunction and TT-a’ which only starts to diminish at more severe degrees of diastolic dysfunction (Additional file 1: Figure S1 and S2). This linear relationship indicates the value of total average diastolic LD as an indicator of LV diastolic function and a total average diastolic LD of 10 mm was found to be a consistent threshold for the general discrimination of patients with or without diastolic dysfunction. This threshold was chosen on the basis of findings in Fig. 3 where the vast majority of controls with normal diastolic function had a total average diastolic LD above 10 mm. Pearson correlation was calculated to be −0.61 with a *p-value* <0.001. Using linear regression, total average diastolic LD was estimated to fall by 2.4 mm for every increase in graded severity of diastolic dysfunction (β = −0.61, *p-value* <0.001). A similar relationship was found when investigating total average diastolic velocity (e’ + a’) and degree of diastolic function (Pearson correlation 0.63, *p-value* <0.001), although a cutoff of 12 cm/s is appropriate here (Additional file 1: Figure S3).

### Total average diastolic longitudinal displacement in various heart conditions

Figure 3 illustrates findings of total average diastolic LD in various heart conditions in comparison to the degree of diastolic dysfunction. Median values of all participants classified as having normal diastolic function had a total average diastolic LD above 10 mm, except for patients with severe systolic heart failure. A closer examination of the patients with severe systolic heart failure with normal diastolic function as determined by current guidelines [2] revealed that patients with a diastolic LD ≥10 mm had reduced markers of systolic function (mean LS = −11.5 ± 7.1 %; mean LSR = −0.85 ± 0.2 s^−1^; and mean systolic LD by TDI (TT-s’) = 10.7 ± 1.5 mm). Systolic function of individuals with severe systolic heart failure, normal diastolic function, and a diastolic LD <10 mm was also reduced, but to a greater degree (LS = −9.9 ± 5.7 %, LSR = −0.68 ± 0.32 s^−1^, TT-s’ = 7.0 ± 1.7 mm).

Generally, median values of mean diastolic LD fell with increasing severity of diastolic dysfunction for all subgroups except for the control group and patients with LV hypertrophy, who both had a preserved total average diastolic LD ≥10 mm despite being categorised as having an abnormal relaxation pattern. Median values of all participants classified as having either a pseudonormal or restrictive filling pattern had a total average diastolic LD <10 mm. Table 3 shows the configuration of the E- and A-waves in controls and in patients with LV hypertrophy and an abnormal relaxation pattern. In both groups, peak velocities for the A-wave were greater than for the E-wave but displacement in the E- and A-waves for each was roughly equal, indicating that these two groups had low, broad E-waves and tall, narrow A-waves.

**Table 3:** The configuration of the early and atrial waves in controls and in subjects with left ventricular hypertrophy and an abnormal relaxation pattern

Configuration	Controls Mean ± SD	LV hypertrophy Mean ± SD
e’ (cm/s)	5.44 ± 1.46	5.11 ± 1.56
a’ (cm/s)	7.27 ± 2.41	7.86 ± 1.22
TT-e’ (mm)	5.44 ± 1.20	5.02 ± 1.51
TT-a’ (mm)	5.31 ± 1.71	6.24 ± 0.91

*e’* early diastolic tissue velocity by TDI measured at the mitral annulus, *a’* late diastolic tissue velocity by TDI measured at the mitral annulus, *TT-e’* early diastolic displacement measured at the mitral annulus, *TT-a’* late diastolic displacement measured at the mitral annulus

A further analysis of controls with grade 1 diastolic dysfunction and a diastolic LD ≥10 mm revealed that the sole reason for these individuals being classified as having grade 1 diastolic dysfunction was a lateral e’ <10 cm/s or septal e’ <8 cm/s and not enlarged atriums (LA volume <34 ml/m^2^).

Table 4 shows the correlation of average diastolic and systolic LD measures with peak average diastolic and systolic velocities for subjects with LV hypertrophy and varying degrees of diastolic dysfunction. A moderate correlation was found between total average systolic LD and total average diastolic LD as well as between peak longitudinal systolic velocity by TDI (s’) and total average diastolic LD, especially in patients with normal diastolic function and in patients with abnormal relaxation. An examination of parameters of systolic function in patients with LV hypertrophy indicates preserved systolic function (LVEF = 60 ± 7 %, LSR = −0.92 ± 0.35 s^−1^, LS = 12.0 ± 3.8, TT-s’ = 10.8 ± 2.0 mm, s’ = 5.38 ± 1.14 cm/s).

**Table 4:** Correlations of total average diastolic longitudinal displacement with peak average diastolic velocities and average systolic longitudinal displacement for subjects with LV hypertrophy and no diastolic dysfunction and a first and second degree of diastolic dysfunction

Comparison	DDF 0		DDF 1		DDF 2	
	Pearson Correlation	*P-value*	Pearson Correlation	*P-value*	Pearson Correlation	*P-value*
e’ and TT-e’	0.92	<0.001	0.77	<0.001	0.67	<0.05
a’ and TT-a’	0.89	<0.001	0.81	<0.001	0.88	<0.001
e’ + a’ and TT-e’ + TT-a’	0.81	<0.001	0.81	<0.001	0.88	<0.001
TT-e’/TT-a’ and e’/a’	0.95	<0.001	0.75	<0.001	0.76	<0.001
TT-s’ and TT-e’ + TT-a’	0.53	<0.05	0.62	<0.05	0.79	<0.001
TT-e’ + TT-a’ and s’	0.46	0.06	0.40	0.09	0.80	<0.001

*e’* early diastolic tissue velocity by TDI measured at the mitral annulus, *a’* late diastolic tissue velocity by TDI measured at the mitral annulus, *s’* systolic tissue velocity by TDI measured at the mitral annulus, *TT-e’* early diastolic displacement measured at the mitral annulus, *TT-a’* late diastolic displacement measured at the mitral annulus, *TT-s’* systolic displacement measured at the mitral annulus

### Left ventricular ejection fraction and total average diastolic longitudinal displacement

**Fig. 4:**
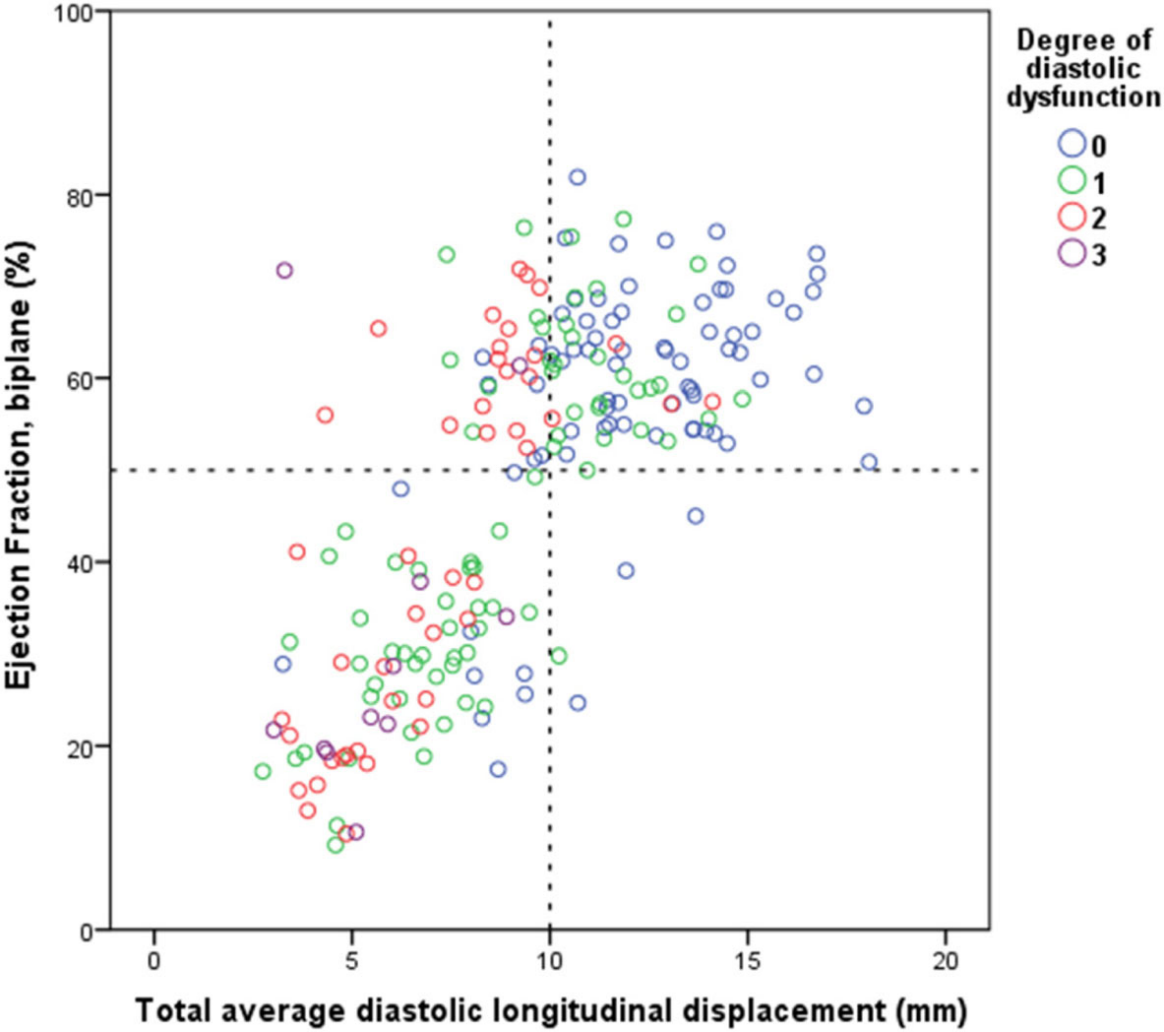
Total average diastolic longitudinal displacement as a function of left ventricular ejection fraction

Figure 4 illustrates the relationship of total average diastolic LD with LVEF. Participants are clustered into two main groups: a group with a total average diastolic LD <10 mm and a LVEF <50 % and a group with a total average diastolic LD >10 mm and a LVEF >50 %, and two smaller groups: a group with total average diastolic LD <10 mm and an LVEF >50 % and a group with a total average diastolic LD >10 mm and an LVEF <50 %.

By examining the colour combinations of subjects in Fig. 4, it can be seen that the vast majority of subjects with no diastolic dysfunction have a LVEF >50 % and a diastolic LD >10 mm while the vast majority of subjects with grade 3 diastolic dysfunction have a LVEF <50 % and a diastolic LD <10 mm.

Regarding subjects with a LVEF >50 % and a diastolic LD <10 mm, it can be seen that almost all subjects have some degree of diastolic dysfunction. A comparison of longitudinal systolic function of these subjects, i.e. patients with LVEF >50 % and diastolic LD <10 mm (n = 32), compared to subjects with LVEF >50 % and diastolic LD >10 mm (n = 88) revealed that systolic function was reduced in this group: LS = −12.3 ± 4.1 %, LSR = −0.86 ± 0.33 s^−1^, TT-s’ = 9.2 ± 1.8 mm and s’ = 4.8 ± 1.0 cm/s compared to LS = −13.9 ± 3.9 %, LSR = −1.05 ± 0.32 s^−1^, TT-s’ = 11.9 ± 2.2 mm and s’ = 6.3 ± 1.4 cm/s.

An examination of systolic function of the 4 individuals with a LVEF <50 % and a diastolic LD >10 mm revealed a LS of −11.5 ± 7.1 %, LSR = −0.78 ± 0.17 s^−1^, TT-s’ = 10.8 ± 1.5 mm and s’ = 5.3 ± 1.2 cm/s. An analysis of the comorbidities of these patients revealed that they all suffered from ischemic heart disease.

### Correlations

Pearsons correlation was used to determine the association of average diastolic LD with a variety of other echocardiographic parameters (Table 5). With regards to the entire population, strong associations were found when comparing LD in early diastole (TT-e’) with early mitral annular velocity (e’), LD in late diastole (TT-a’) with late mitral annular velocity (a’), total average diastolic LD (TT-e’ + TT-a’) with e’ + a’, and the ratio of diastolic LD between the early and atrial wave (TT-e’/TT-a’) with peak velocities for the early and atrial wave (e’/a’). When considering systolic displacement measured by TDI, M-mode, and 2D strain imaging and TDI derived total average diastolic LD, strong correlations were found. Weaker correlations were found when comparing total average diastolic LD to other systolic measures, such as LVEF, LS and LSR. A comparison of associations between groups (Table 5) reveals that the strongest associations were seen when comparing diastolic LD measurements with diastolic velocity measurements. A weaker association was seen when comparing average diastolic LD with systolic LD and velocity, and was weakest for patients with LV hypertrophy. The biggest fluctuations and weakest correlations between groups were seen when determining the association of average diastolic LD with markers of systolic function such as LVEF, LS and LSR.

**Table 5:** Correlations of average diastolic longitudinal displacement with peak average diastolic velocities, average systolic longitudinal displacement and other measures of systolic function for all subjects and between subgroups

Comparison	All subjects		Control		Severe systolic HF		LV hypertrophy		LV dilation		Mitral regurgitation	
	r	*p-value*	r	*p-value*	r	*p-value*	r	*p-value*	r	*p-value*	r	*p-value*
e’ and TT-e’	0.93	<0.001	0.90	<0.001	0.89	<0.001	0.88	<0.001	0.91	<0.001	0.95	<0.001
a’ and TT-a’	0.92	<0.001	0.87	<0.001	0.94	<0.001	0.89	<0.001	0.90	<0.001	0.92	<0.001
e’ + a’ and TT-e’ + TT-a’	0.94	<0.001	0.84	<0.001	0.89	<0.001	0.85	<0.001	0.88	<0.001	0.96	<0.001
TT-e’/TT-a’ and e’/a’	0.90	<0.001	0.92	<0.001	0.87	<0.001	0.94	<0.001	0.94	<0.001	0.86	<0.001
TT-e’/TT-a’ and E/A	0.67	<0.001	0.83	<0.001	0.79	<0.001	0.87	<0.001	0.52	<0.001	0.62	<0.001
TT-s’ and TT-e’ + TT-a’	0.88	<0.001	0.82	<0.001	0.73	<0.001	0.66	<0.001	0.76	<0.001	0.88	<0.001
s’ and TT-e’ + TT-a’	0.85	<0.001	0.73	<0.001	0.70	<0.001	0.56	<0.001	0.78	<0.001	0.89	<0.001
e’ + a’ and s’	0.82	<0.001	0.74	<0.001	0.74	<0.001	0.43	<0.001	0.75	<0.001	0.87	<0.001
LVEF and TT-e’ + TT-a’	0.71	<0.001	−0.03	0.83	0.52	<0.001	0.01	0.94	0.81	<0.001	0.58	<0.001
LS and TT-e’ + TT-a’	−0.60	<0.001	−0.06	0.67	−0.50	<0.001	−0.18	0.21	−0.57	<0.001	−0.64	<0.001
LSR and TT-e’ + TT-a’	−0.41	<0.001	0.05	0.73	−0.29	0.05	−0.31	<0.05	−0.40	<0.05	−0.52	<0.001
LD and TT-e’ + TT-a’	0.76	<0.001	0.52	<0.001	0.31	<0.05	0.33	<0.05	0.75	<0.001	0.78	<0.001
MAPSE and TT-e’ + TT-a’	0.82	<0.001	0.63	<0.001	0.54	<0.001	0.37	<0.01	0.83	<0.001	0.84	<0.001

*HF* heart failure, *LV* left ventricular, *e’* early mitral annular diastolic velocity, *TT-e’* displacement in early diastole, *a’* late mitral annular diastolic velocity, *TT-a’* displacement in late diastole, *TT-e’ + TT-a’* total average diastolic displacement; s’, mitral annular systolic velocity, *TT-s’* total average systolic displacement, *LVEF* left ventricular ejection fraction, *LS* longitudinal strain derived from the 4-chamber view, *LSR* longitudinal strain rate derived from the 4-chamber view, *LD* longitudinal displacement derived from strain imaging in the 4-chamber view, *MAPSE* M-mode derived mitral annular plane systolic excursion

### Agreement with echocardiographic parameters

**Fig. 5:**
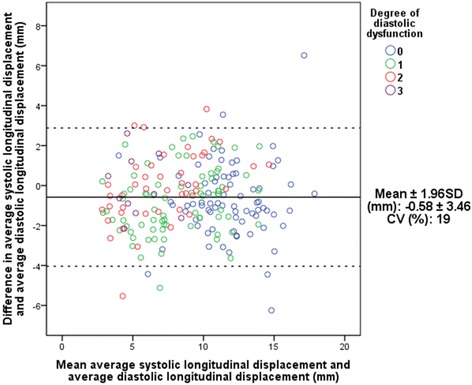
Interchangeability of average systolic displacement and total average diastolic longitudinal displacement. *CV, coefficient of variation; SD, standard deviation*

Figure 5 depicts the mean differences (bias) of total average systolic and diastolic measurements plotted against the mean of the two average LD measurements, (Bland-Altman plot). The mean difference was calculated to −0.58 mm indicating that average systolic LD underestimates total average diastolic LD. The limits of agreement were calculated to ±3.46 mm and a coefficient of variation (CV) of 19 % was found.

### Total average diastolic longitudinal displacement as a predictor of adverse cardiac outcomes

**Fig. 6:**
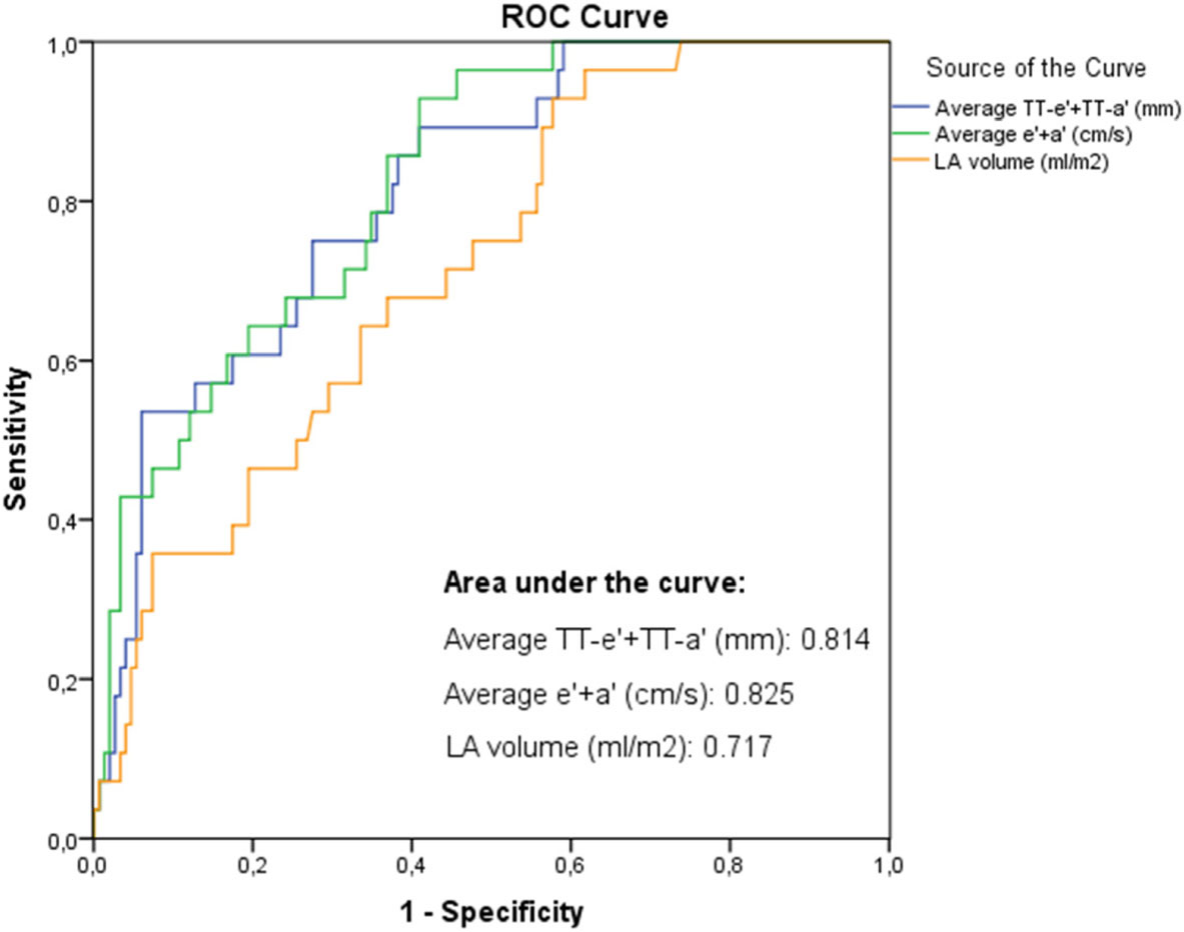
Roc curves illustrating a parameter’s predictive value of death. *e’, early diastolic tissue velocity by TDI measured at the mitral annulus; a’, late diastolic tissue velocity by TDI measured at the mitral annulus; TT-e’, early diastolic displacement measured at the mitral annulus; TT-a’, late diastolic displacement measured at the mitral annulus; LA, left atrial*

There were 33 events (death) during 2.6 ± 1.2 years follow-up. Odds ratios were calculated to determine the predictive capabilities of total diastolic LD in comparison to diastolic colour TDI velocities with regards to all-cause mortality: per unit decrease, risk of death increased in a similar pattern for e’ + a’ and TT-e’ + TT-a’ (Table 6). Following adjustment for covariates (first multivariate analysis adjusted for age and gender and second multivariate analysis adjusted for age, gender, LVEF, LV internal dimension in diastole/body surface area, LV mass index, and LS), e’ + a’ and TT-e’ + TT-a’ remained significant predictors of death. LA volume was also shown to be a significant predictor of death, also after adjustment in multivariate analyses. The calculation of area under the curve in ROC curves (Fig. 6) showed similar results.

**Table 6:** Unadjusted and adjusted univariable and multivariable odds ratios

	Unadjusted model:		Multivariable model adjusted for age and gender:		Multivariable model adjusted for age, gender, LVEF, LVIDd/BSA, LVMI, LS:	
	Odds ratio (95 % CI)	*P-value*	Odds ratio (95 % CI)	*P-value*	Odds ratio (95 % CI)	*P-value*
TT-e’ + TT-a’ per 1 mm decrease	1.47 (1.26–1.71)	*<0.001*	1.33 (1.13–1.57)	*<0.01*	1.35 (1.04–1.74)	*<0.05*
TT-e’ per 1 mm decrease	1.63 (1.30–2.04)	*<0.001*	1.39 (1.07–1.80)	*<0.05*	1.18 (0.82–1.69)	*0.374*
TT-a’ per 1 mm decrease^a^	1.59 (1.26–2.03)	*<0.001*	1.51 (1.16–1.95)	*<0.01*	1.38 (1.01–1.88)	*<0.05*
TT-s’ per 1 mm decrease	1.31 (1.16–1.48)	*<0.001*	1.18 (1.03–1.36)	*<0.05*	1.05 (0.83–1.33)	*0.703*
-e’ per 1 cm/s decrease	1.70 (1.34–2.15)	*<0.001*	1.41 (1.07–1.85)	*<0.05*	1.24 (0.90–1.72)	*0.192*
-a’ per 1 cm/s decrease^a^	1.45 (1.20–1.73)	*<0.001*	1.35 (1.10–1.65)	*<0.01*	1.25 (0.99–1.58)	*0.066*
-e’ + a’ per 1 cm/s decrease	1.42 (1.24–1.62)	*<0.001*	1.30 (1.12–1.51)	*<0.01*	1.27 (1.04–1.55)	*<0.05*
Per degree of DDF:						
• No DDF	-	*-*	-	*-*	-	*-*
• DDF 1	2.26 (0.80–6.38)	*0.124*	0.76 (0.22–2.58)	*0.658*	0.39 (0.10–1.50)	*0.171*
• DDF 2	5.44 (1.91–15.52)	*<0.01*	1.59 (0.48–5.26)	*0.451*	0.80 (0.21–3.02)	*0.744*
• DDF 3	1.17 (0.13–10.72)	*0.892*	0.59 (0.06–5.94)	*0.652*	0.21 (0.02–2.51)	*0.216*
E/e’ per 1 unit decrease	1.09 (1.04–1.14)	*<0.001*	1.05 (1.01–1.09)	*<0.05*	1.04 (1.00–1.09)	*0.058*
E/A per 1 unit decrease	1.04 (0.61–1.79)	*0.881*	1.43 (0.78–2.60)	*0.248*	1.28 (0.69–2.37)	*0.432*
DT per 1 ms decrease	1.00 (1.00–1.01)	*0.921*	1.00 (0.99–1.00)	*0.535*	1.00 (1.00–1.01)	*0.997*
LAD per 1 cm decrease	1.56 (0.53–4.63)	*0.420*	1.85 (0.42–8.19)	*0.420*	-	*-*
LA volume per 1 ml/m^2^ decrease	1.05 (1.02–1.08)	*<0.001*	1.04 (1.01–1.07)	*<0.05*	1.05 (1.01–1.09)	*<0.05*
Per change in patient subgroup:						
• Control	-	*-*	-	*-*	-	*-*
• Cardiac dysfunction	9.08 (1.07–77.08)	*<0.05*	3.75 (0.39–36.30)	*0.253*	1.01 (0.07–15.01)	*0.993*
• LV hypertrophy	5.46 (0.61–48.53)	*0.128*	1.89 (0.19–18.79)	*0.586*	1.06 (0.09–12.34)	*0.966*
• LV dilation	27.79 (3.35–230.35)	*<0.01*	7.49 (0.83–67.96)	*0.073*	0.75 (0.03–18.18)	*0.859*
• Mitral regurgitation	18 (2.15–150.46)	*<0.01*	7.19 (0.79–65.87)	*0.081*	3.88 (0.39–38.60)	*0.248*

*DDF* Degree of diastolic dysfunction, *e’* early mitral annular diastolic velocity, *TT-e’* displacement in early diastole, *a’* late mitral annular diastolic velocity, *TT-a’* displacement in late diastole, *TT-e’ + TT-a’* total average diastolic displacement, *s’* mitral annular systolic velocity, *TT-s’* total average systolic displacement, *DT* deceleration time, *LAD* left atrial diameter, *LA* left atrial

^a^Interaction with subgroup type

### Reproducibility

Intraobserver variability for septal and lateral LD measurements of the E-wave and A-wave was relatively low, as is shown by the mean difference being close to zero (Additional file 1: Figure S4). Limits of agreement are, however, fairly wide and the largest variability, as indicated by CV, was seen in lateral A-wave LD measurements.

Interobserver variability findings were found to be similar to intraobserver variability. All mean differences were close to zero. The greatest variability was seen in E-wave LD measurements in comparison to A-wave LD measurements, as is indicated by the larger limits of agreement and larger CV.

## Discussion

This is the first study, to our knowledge, to investigate total average diastolic LD determined by colour TDI as a potential new parameter for the determination of LV diastolic dysfunction. Our findings on the predictive capabilities and reproducibility of this parameter are important with regards to its utilisation in clinical practice.

### Tissue Doppler Imaging

TDI is a useful echocardiographic technique to evaluate global and regional systolic and diastolic function in a variety of different cardiac conditions [14]. Even though it is reproducible, widespread, and allows for extensive off-line analysis [15], it is underutilised in clinical practice. Limitations of TDI include angle dependency with possible underestimations of tissue velocities if the angle of interrogation exceeds 20° [14] and overestimations when using excessive gain [16]. Furthermore, difficulties in estimating diastolic tissue velocities and displacement arise in patients with non-discernible E and A waves, i.e. patients with atrial fibrillation.

### Total average diastolic longitudinal displacement and degree of diastolic dysfunction

One of the major drawbacks of current diastolic parameters is that there often is a biphasic element involved, as is seen with E/A and DT. This results in difficulty in discerning between a patient with normal diastolic function and a patient with a relatively severe grade of diastolic dysfunction. As depicted in Fig. 2, total average diastolic LD has a linear relationship with the degree of diastolic dysfunction, thereby eliminating this risk of misinterpretation. This linear relationship is due to a fall in TT-e’ in the mild stages of diastolic dysfunction and TT-a’ which only starts to diminish at more severe degrees of diastolic dysfunction (Additional file 1: Figure S1 and S2). A total average diastolic LD of 10 mm was found to be a consistent threshold for the general discrimination of patients with or without diastolic dysfunction and this can potentially be used in a clinical situation as an independent marker for the general evaluation of a patient’s overall diastolic function. Furthermore, total average diastolic LD considers the shape of the E- and A-wave and not just peak velocity values. Theoretically, this must provide a more reliable measurement in comparison to single snapshot based parameters, see below.

### Total average diastolic longitudinal displacement in various heart conditions

Total average diastolic LD is a potentially superior parameter for the true evaluation of diastolic dysfunction in comparison to current velocity based echocardiographic parameters: total average diastolic LD looks at the entire diastolic process as it takes the shape of the E and A diastolic waves, and not just peak values, into consideration. This is illustrated in Fig. 3 where controls and patients with LV hypertrophy have a preserved total average diastolic dysfunction despite being classified as having abnormal relaxation. A compensatory mechanism that is not measured by current diastolic evaluation methods could, potentially, be at work. An analysis of the configuration of the E- and A-waves in controls and in patients with LV hypertrophy and an abnormal relaxation pattern (Table 3) reveals that preserved diastolic LD is not due to a compensatory increase in overall A-wave size, as one might anticipate, but is due to E-waves being low and broad in shape and A-waves being high and narrow in shape, a situation that is not taken into consideration when just looking at peak velocities.

An analysis of the various heart conditions of the patients included in this study reveals that total average diastolic LD is a potential indicator of diastolic dysfunction irrespective of the primary heart condition, as discussed below:

With regards to the control group, as expected, the majority of patients are categorised as having either normal diastolic function or grade 1 diastolic dysfunction. An unexpected finding, however, is that the majority of control patients with grade 1 diastolic dysfunction have a diastolic LD >10 mm. On further analysis, these individuals did not have enlarged left atriums and the sole reason for these individuals being classified as having grade 1 diastolic dysfunction was a LA volume <34 ml/m^2^. The majority of these patients also had an E/A ratio <0.8.

With regard to patients with severe systolic heart failure, it is questionable how a patient with severely reduced systolic function (eyeball LVEF <30 %) can have a preserved diastolic function as, with regards to conventional understanding, a reduced systolic function should result in a reduced diastolic function and vice versa. A closer inspection of these patients revealed that the individuals with a diastolic LD >10 mm had reduced markers of systolic function, though greater than individuals with a diastolic LD <10 mm.

With regards to patients with LV hypertrophy, a preserved total average diastolic LD is seen despite patients being categorised as having an abnormal relaxation pattern, indicating that total average diastolic LD may not be optimal for the discernment of diastolic dysfunction in this patient group. As described above, an analysis of the E- and A-waves of these patients (Table 3 *)* reveals broad E-waves and tall, narrow A-waves. For subjects with LV hypertrophy and grade 1 and 2 diastolic dysfunction (Table 4), a moderate correlation was found between total average systolic LD and total average diastolic LD as well as between s’ and total average diastolic LD indicating a potentially preserved systolic function and reduced diastolic function, as would be expected in patients with LV hypertrophy. An examination of parameters of systolic function indicates slightly reduced systolic function.

With regards to patients with LV dilation and mitral regurgitation, the expected pattern of falling diastolic LD with increasing grades of diastolic dysfunction is seen. No patients with LV dilation have normal diastolic function, as is to be expected.

### Systolic and diastolic function

LV systolic dysfunction is commonly differentiated from LV diastolic dysfunction by the presence of a reduced LVEF [17, 18]. This differentiation is potentially erroneous as abnormalities of contractile function and diastolic dysfunction have been shown to coexist, and diastolic dysfunction often has its genesis in systole [19]. The idea of isolated diastolic function can, therefore, be questioned and it can be suggested that heart failure should be viewed as an individual disease where systolic and diastolic dysfunction are the extremes on a spectrum of different phenotypes of the same disease [19, 20]. In this study, systolic and diastolic function are shown to go hand-in-hand, i.e. a patient with poor systolic function is also shown to have poor diastolic function. This relationship is clearly depicted when using total average diastolic LD as a marker of diastolic dysfunction (Fig. 4), indicating that LD is a potentially valid substitute for present velocity-based determinations of diastolic dysfunction.

Likewise, it is relevant to test the correlation between average systolic LD and total average diastolic LD as, intuitively, heart LD in systole must equal heart LD in diastole if the heart is to remain the same overall size. Good associations were found with regards to total average diastolic LD and measurements of systolic heart function such as peak systolic velocity (s’), systolic LD (TT-s’), M-mode derived MAPSE and 2D strain derived LD (Table 5). This finding, as well as the relationship seen between LVEF and total average diastolic dysfunction in Fig. 4, also challenges the concept of isolated diastolic dysfunction. It would be correct to classify patients with reduced diastolic function and preserved LVEF as patients with heart failure with preserved ejection fraction (HFPEF) but incorrect to assume that these patients have isolated diastolic dysfunction as we found that systolic function, determined by longitudinal deformation measurements such as s’ and LS, was reduced in these patients despite a preserved LVEF. This indicates that diastolic LD may additionally be a marker of systolic function that is more sensitive than LVEF.

Despite acceptable correlations between total average diastolic LD and measurements of systolic heart function (Table 5), it can be seen that echocardiographic systolic measurements are not identical to diastolic measurements. This may be partially due to the presence of L-waves which were not included in the assessment of diastolic LD as only E- and A-waves were evaluated. L-waves are often observed in mid-diastole and indicate continued pulmonary vein flow through the left atrium into the left ventricle after the E-wave [21]. Furthermore, the discrepancy between total average diastolic LD and systolic heart function may indicate that the relationship between early and late diastole may reveal aspects of heart pump function that cannot be revealed by an assessment of systolic function alone.

### Interchangeability and reproducibility of displacement measurements

Average systolic LD was found to slightly underestimate total average diastolic LD by approximately 0.6 mm (Fig. 5). Even though limits of agreement are wide (±3.46 mm), a CV of 19 % suggests that these two parameters are potentially interchangeable.

Furthermore, as is shown in Table 5, the correlation between average diastolic peak velocity measurements and total average diastolic LD was very good and a similar relationship of e’ + a’ with the degree of diastolic dysfunction compared to TT-e’ + TT-a’ and the degree of diastolic function was found, albeit a cut-off of 12 cm/s is more appropriate here instead of 10 mm (Additional file 1: Figure S3). Furthermore, area under the curve illustrating the predictive value of TT-e’ + TT-a’ and e’ + a’ was very similar. As total average diastolic LD is a little tricky and time consuming to measure, average diastolic peak velocity measurements seems to be a valid substitute.

Reproducibility of displacement measurements of E- and A-waves was reasonable, taking into consideration that this is the first time that diastolic LD by colour TDI has been validated, and was fairly equal in the determination of both intra and interobserver variability. Limits of agreement were fairly wide and the largest variability was seen in the determination of displacement of the lateral A-wave.

### Prediction of adverse cardiac outcomes

An analysis of death as an endpoint, using odds ratios and ROC curve analyses, showed that risk of death per unit decrease in both average diastolic LD (TT-e’ + TT-a’) and average peak diastolic velocities (e’ + a’) was similar, even after adjustment for covariates.

### Clinical implications

Total average diastolic LD has the potential to revolutionise the way in which we look at diastolic function. It looks at the entire diastolic process, therefore, making it a promising and sensitive marker of diastolic function. Furthermore, it has the potential to be used in a clinical setting as a quick and effective determinant of patients with and without diastolic dysfunction.

### Study limitations

A variety of limitations in this study have to be addressed:

Firstly, the validity of total average diastolic LD was measured against determinants from current guidelines for the non-invasive determination of diastolic dysfunction as the golden standard. Future research with the use of invasive diastolic measurements is, therefore, required.

Secondly, only the septal and lateral wall in the 4-chamber view was assessed. A truer (but also much more time-consuming) depiction of global longitudinal diastolic performance would be obtained if the anterior, inferior, posterior and anteroseptal mitral annular sites were included.

Thirdly, all-cause mortality was the only primary endpoint evaluated in this study. An assessment of more endpoints, including cardiovascular death, may show the prognostic value of total average diastolic LD to be even higher.

## Conclusions

Total average diastolic LD is a promising alternative to current algorithm-based evaluations. It has a linear relationship with the degree of diastolic dysfunction assessed by current guidelines and a 10 mm threshold in total average diastolic LD can potentially be used independently to evaluate patients with and without diastolic dysfunction in a variety of different heart conditions with reasonable intra and interobserver reproducibility.

## Additional file

Additional file 1: Figure S1. Mean early diastolic longitudinal diaplacement velocity and degree of diastolic dysfunction. **Figure S2.** Mean atrial diastolic longitudinal diaplacement velocity and degree of diastolic dysfunction. **Figure S3.** Total average diastolic velocity and degree of diastolic dysfunction. Dotted line at 12 cm/s represents cutoff to discern normal diastolic function from diastolic dysfunction. **Figure S4.** Reproducibility of early and late diastolic displacement measurements. (DOCX 313 kb)

## Abbreviations

2DSI: Two-dimensional strain imaging
A: Atrial mitral inflow velocity
a’: Atrial mitral annular diastolic velocity
A-wave: Atrial mitral annular diastolic velocity wave
CV: Coefficient of variation
DT: Deceleration time
E: Early mitral inflow velocity
e’: Early mitral annular diastolic velocity
E-wave: Early mitral annular diastolic velocity wave
LA: Left atrial
LD: Longitudinal displacement
LS: Longitudinal strain
LSR: Longitudinal strain rate
LV: Left ventricle
LVEF: Left ventricular ejection fraction
TDI: Tissue Doppler imaging
TT-a’: Mean atrial diastolic longitudinal displacement
TT-e’: Mean early diastolic longitudinal displacement
TT-e’ + TT-a’: Total average diastolic longitudinal displacement
TT-s’: Mean systolic longitudinal displacement
