# Supplementary material for: Total average diastolic longitudinal displacement by colour tissue doppler imaging as an assessment of diastolic function
Source: Cardiovasc Ultrasound. 2016 Sep 17;14:41. doi: 10.1186/s12947-016-0083-2 (PMC5027100; doi:10.1186/s12947-016-0083-2)
Supplement: Additional file 1: Figure S1. — Mean early diastolic longitudinal diaplacement velocity and degree of diastolic dysfunction. Figure S2. Mean atrial diastolic longitudinal diaplacement velocity and degree of diastolic dysfunction. Figure S3. Total average diastolic velocity and degree of diastolic dysfunction. Dotted line at 12 cm/s represents cutoff to discern normal diastolic function from diastolic dysfunction. Figure S4. Reproducibility of early and late diastolic displacement measurements. (DOCX 313 kb) [file 12947_2016_83_MOESM1_ESM.docx]

**Additional file 1**

**Figure S1**. Mean early diastolic longitudinal diaplacement velocity and degree of diastolic dysfunction.


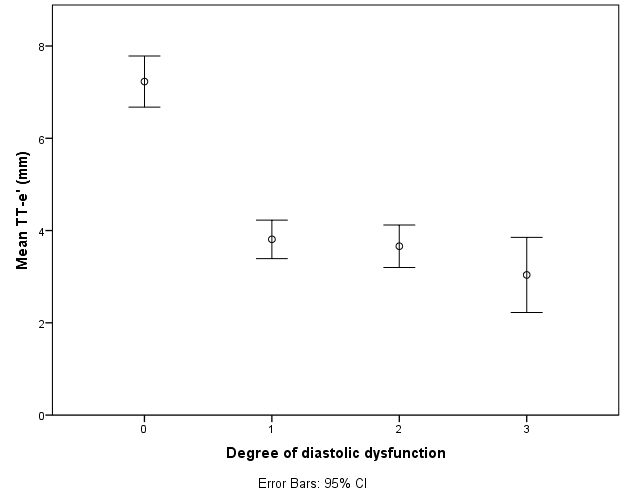


*TT-e’, early diastolic longitudinal displacement*

**Figure S2**. Mean atrial diastolic longitudinal diaplacement velocity and degree of diastolic dysfunction.


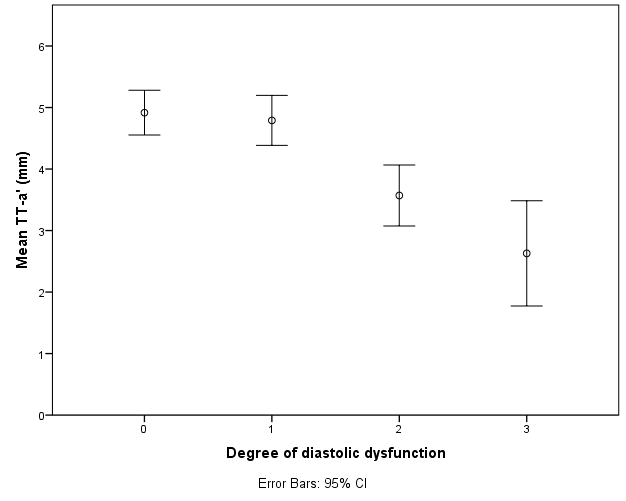


*TT-a’, atrial diastolic longitudinal dispalcement*

**Figure S3.** Total average diastolic velocity and degree of diastolic dysfunction. Dotted line at 12 cm/s represents cutoff to discern normal diastolic function from diastolic dysfunction.


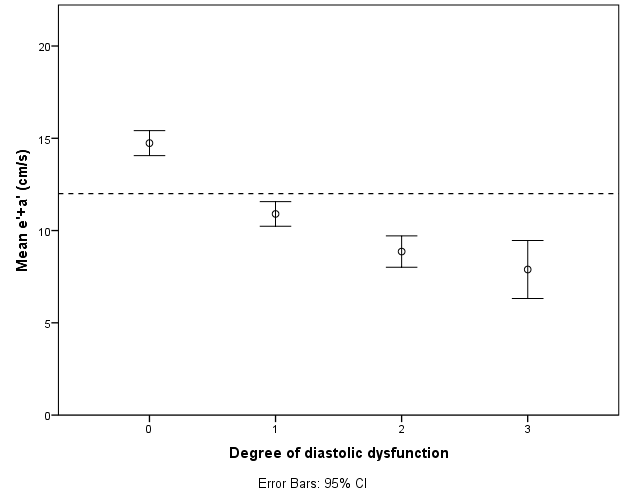


*e’, early mitral annular diastolic velocity; a’, atrial mitral annular diastolic velocity*

**Figure S4.** Reproducibility of early and late diastolic displacement measurements.

**
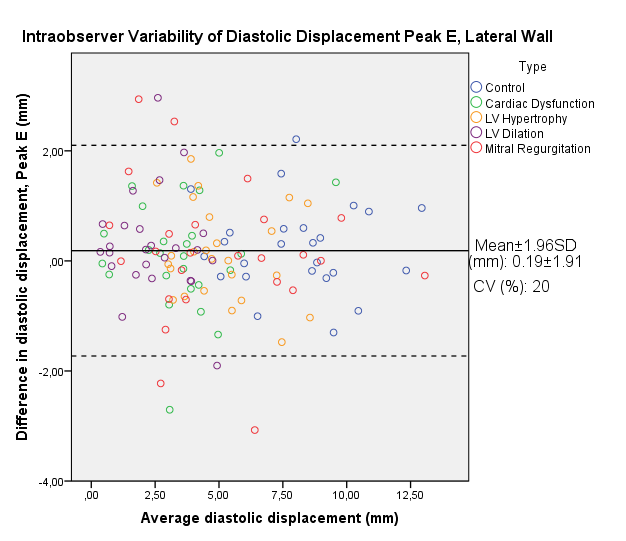

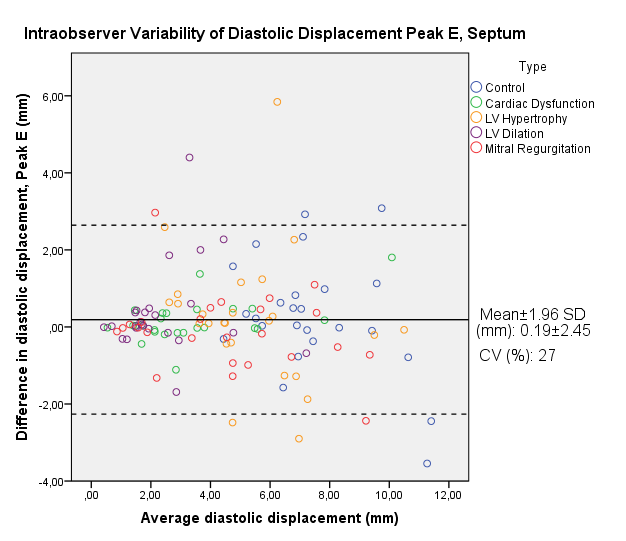
**

**
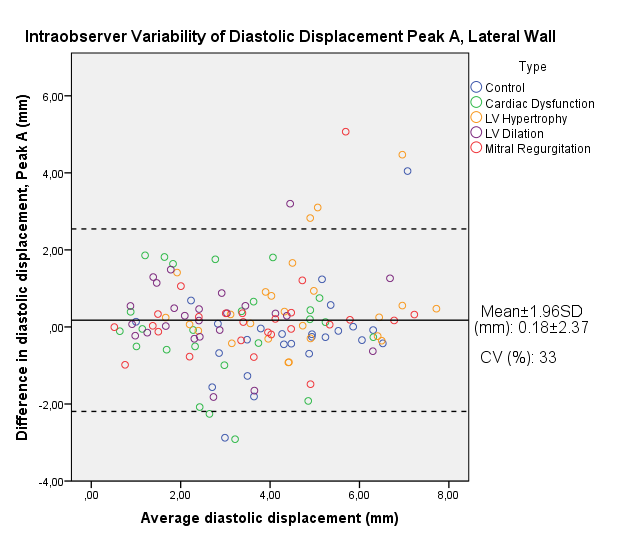

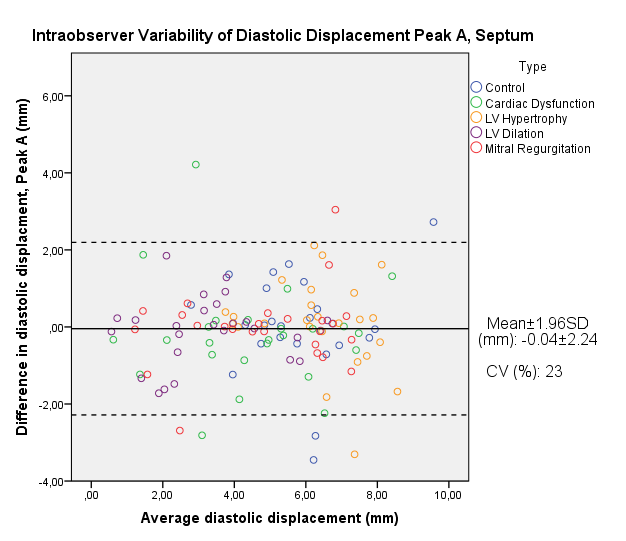
**

**
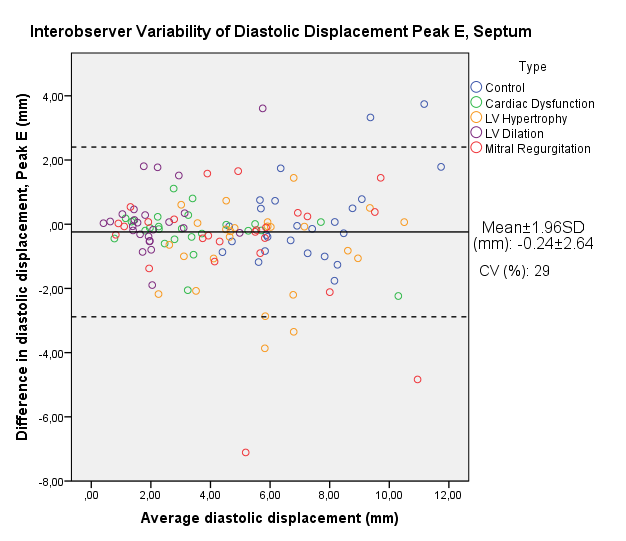

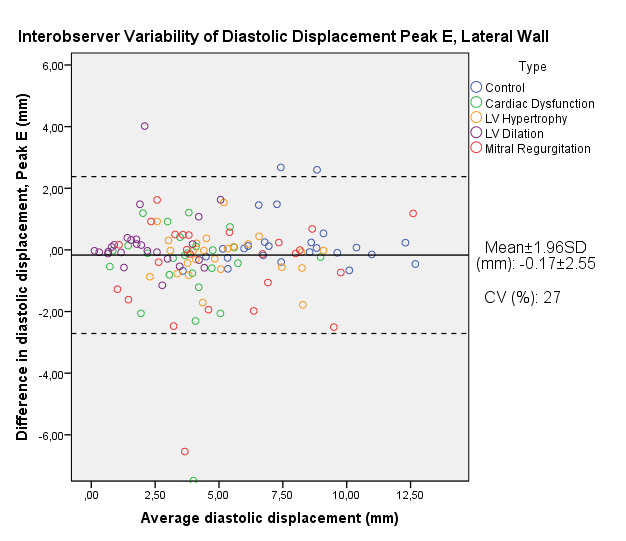

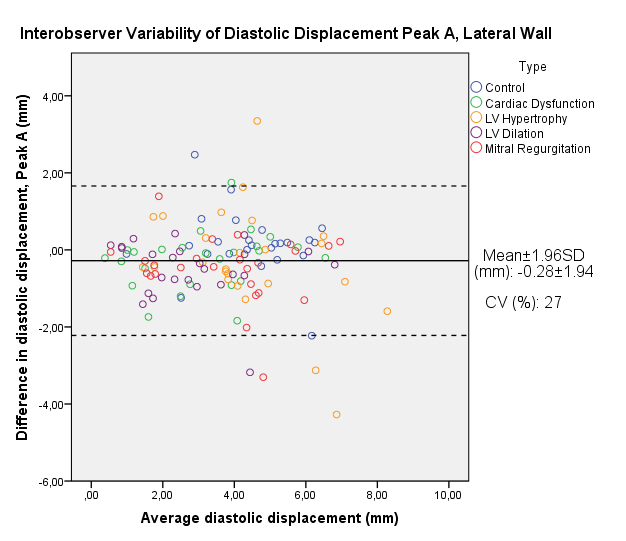

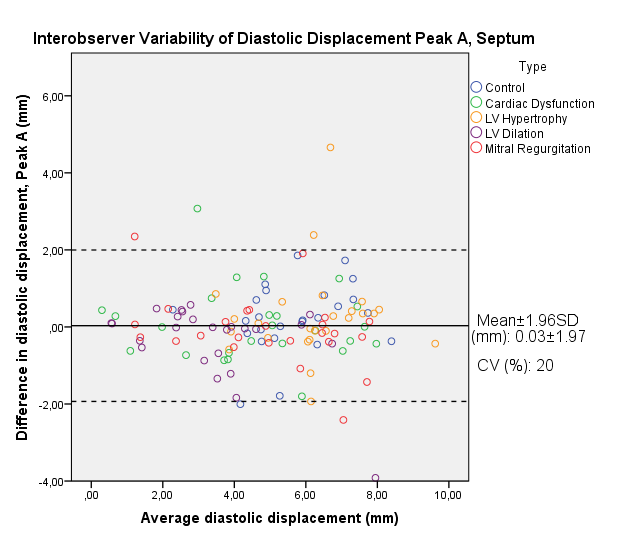
**
